# Temporal trends in childhood mortality in Ghana: impacts and challenges of health policies and programs

**DOI:** 10.3402/gha.v9.31907

**Published:** 2016-08-23

**Authors:** Gbenga A. Kayode, Diederick E. Grobbee, Augustina Koduah, Mary Amoakoh-Coleman, Irene A. Agyepong, Evelyn Ansah, Han van Dijk, Kerstin Klipstein-Grobusch

**Affiliations:** 1 Julius Global Health, Julius Center for Health Sciences and Primary Care, University Medical Centre Utrecht, Utrecht, The Netherlands; 2 Ministry of Health, Accra, Ghana; 3 Social Science Group, Wageningen University and Research Center, Wageningen, The Netherlands; 4 School of Public Health, University of Ghana, Legon, Accra, Ghana; 5 Ghana Health Service, Greater Accra Region, Accra, Ghana; 6 Division of Epidemiology and Biostatistics, School of Public Health, Faculty of Health Sciences, University of the Witwatersrand, Johannesburg, South Africa

**Keywords:** neonatal, infant, under-five mortality, Ghana

## Abstract

**Background:**

Following the adoption of the Millennium Development Goal 4 (MDG 4) in Ghana to reduce under-five mortality by two-thirds between 1990 and 2015, efforts were made towards its attainment. However, impacts and challenges of implemented intervention programs have not been examined to inform implementation of Sustainable Development Goal 3.2 (SDG 3.2) that seeks to end preventable deaths of newborns and children aged under-five. Thus, this study aimed to compare trends in neonatal, infant, and under-five mortality over two decades and to highlight the impacts and challenges of health policies and intervention programs implemented.

**Design:**

Ghana Demographic and Health Survey data (1988–2008) were analyzed using trend analysis. Poisson regression analysis was applied to quantify the incidence rate ratio of the trends. Implemented health policies and intervention programs to reduce childhood mortality in Ghana were reviewed to identify their impact and challenges.

**Results:**

Since 1988, the annual average rate of decline in neonatal, infant, and under-five mortality in Ghana was 0.6, 1.0, and 1.2%, respectively. From 1988 to 1989, neonatal, infant, and under-five mortality declined from 48 to 33 per 1,000, 72 to 58 per 1,000, and 108 to 83 per 1,000, respectively, whereas from 1989 to 2008, neonatal mortality increased by 2 per 1,000 while infant and under-five mortality further declined by 6 per 1,000 and 17 per 1,000, respectively. However, the observed declines were not statistically significant except for under-five mortality; thus, the proportion of infant and under-five mortality attributed to neonatal death has increased. Most intervention programs implemented to address childhood mortality seem not to have been implemented comprehensively.

**Conclusion:**

Progress towards attaining MDG 4 in Ghana was below the targeted rate, particularly for neonatal mortality as most health policies and programs targeted infant and under-five mortality. Implementing neonatal-specific interventions and improving existing programs will be essential to attain SDG 3.2 in Ghana and beyond.

## Introduction

Early childhood mortality continues to remain a prominent global health issue even though Millennium Development Goal 4 (MDG 4) was universally adopted to reduce under-five mortality by two-thirds between 1990 and 2015 (1). Also, several ‘calls for action’ to reduce neonatal mortality have been made (2–5), and in response, both governmental and non-governmental bodies have committed considerable resources to this public health challenge. Similar to other low- and middle-income countries (LMICs), post-adoption of MDG 4 in Ghana has witnessed formulation and implementation of maternal and child health policies and intervention programs towards actualizing MDG 4. For example, from 1988 to 1998, the Safe Motherhood Program (SMP) (6), Life Saving Skills (LSS) program (7), and Integrated Management of Childhood Illness (IMCI) program (8) were initiated. The SMP aims to secure safe delivery for women and improve child health services while the LSS (7) seeks to sharpen the clinical skills of midwives. Similarly, the IMCI (8) targets to improve child survival through the provision of clinical guidelines for management of childhood illnesses, health system strengthening, and improving community health practices. In the subsequent decade, from 1998 to 2008, some additional intervention programs and policies implemented were the Community-Based Health Planning and Services (CHPS) (9), User Fees Exemption for Delivery (UFED) (10), Focused Antenatal Care (FANC) (11), and National Health Insurance Scheme (NHIS) (12). The CHPS program aims to bring healthcare closer to the people through primary health care service while the UFED (10) and NHIS programs seek to ease the financial burden of healthcare service and reduce inequality in healthcare uptake. The FANC pursues improvement in maternal and child survival through individualized antenatal care that entails a comprehensive assessment of pregnant women in terms of their socio-cultural beliefs, lifestyle, and medical characteristics to improve early detection and treatment of illness and pregnancy complications. In addition to these national programs and policies, various regions also implemented different intervention programs, for example, the Kybele program in the Greater Accra region (13, 14), Accelerated Child Survival and Development (ACSD) (15) sponsored by United Nation Children and Education Fund (UNICEF) in the Northern, Upper East, and Upper West regions, and Kangaroo Mother Care (KMC) (16) which commenced in six regions in 2007. Although the deadline for the attainment of MDG 4 has elapsed, 99% of childhood mortality still occurs in LMICs (5, 17), with Africa accounting for about 50% (18). Assessment of progress made so far is of utmost importance to inform policy makers and healthcare planners tasked to realize the newly adopted Sustainable Development Goal 3.2 (SDG 3.2) that seeks to end preventable deaths of newborns and under-five children by 2030.

Thus, this study aimed to 1) compare the temporal trends in neonatal, infant, and under-five mortality in Ghana from 1988 to 2008; 2) describe the trends in the proportion of infant and under-five mortality attributed to neonatal deaths in Ghana over the same period; 3) compare national and regional trends in neonatal mortality over the same period; and 4) identify the impact and challenges of health policies and intervention programs implemented in Ghana during this time period.

## Methods

### Setting

Ghana is located in sub-Saharan Africa, along the Gulf of Guinea with a total population of about 24.4 million (19). It has an annual growth rate of about 2.4% per year (20). Ghana has 10 administrative regions, namely Greater Accra, Western, Central, Volta, Eastern, Ashanti, Brong-Ahafo, Northern, Upper East, and Upper West. It has about 100 ethnic groups with different languages but the major ethnic groups are Akan, Ewe, Mole-Dagbane, Guan, and Ga-Adangbe (21).

### Design of data collection

This longitudinal study compared the trends in neonatal mortality, infant and under-five mortality, and described the trends in the proportion of infant and under-five mortality attributed to neonatal deaths in Ghana, from 1988 to 2008, using Ghana Demographic and Health Survey (GDHS) datasets obtained in 1988, 1993, 1998, 2003, and 2008 (22). These datasets were collected by the ICF Macro in conjunction with the Ghana Statistical Service and the Ministry of Health/Ghana Health Service. All the GDHSs followed the same sampling technique; households were randomly sampled for interview by applying a stratified, two-stage cluster random sampling technique. All women and men in all the selected households, within the age range 15–49 and 15–59 years, respectively, were targeted for face-to-face interview using questionnaires. Prior to the interview, informed consent was obtained from every participant. The datasets are nationally representative with an individual response rate of 95–97% and a household response rate of 97–99%. The datasets were weighted to have a better representation of the study population. In GDHS, neonatal mortality was defined as the probability of dying within the first month of life, infant mortality was defined as the probability of dying before the age of 12 months, and under-five mortality was defined as the probability of dying before the age of 60 months. Detailed information on the sampling techniques and procedures for the data collection has been published elsewhere (22). In order to highlight the impact and challenges of health policies and intervention programs implemented in Ghana from 1988 to 2008, MEDLINE, EMBASE, Google Scholar, African Index Medicus, and Ghana Medical Journal were searched, and the articles that assessed the impact and challenges of these interventions implemented from 1988 to 2008 in Ghana were identified and reviewed.

### Statistical analysis

Neonatal, infant, and under-five mortality rates estimated at national and regional level from each GDHS were used to perform trend analysis. Temporal trend patterns were depicted by plotting the number of neonatal deaths per 1,000 live births against the year when the data were captured; infant and under-five mortality underwent a similar analysis at national level. Also, temporal trend pattern of neonatal mortality at the national level was compared with that of regions. Likewise, the proportion of infant and under-five mortality attributed to neonatal mortality was examined by plotting the percentage of infant and under-five mortality attributed to neonatal death against the year when the data were captured. In order to quantify the trends objectively, a Poisson regression analysis was applied to quantify the incidence rate ratios of the trends. Statistical significance was determined by two-tailed Wald test at significant level of alpha equal to 5%; all analyses were performed in Stata statistical software package version 11 (23).

### Ethical approval

Anonymous publicly available data were utilized in this study. Thus, no ethical approval is required.

## Results

### Descriptive statistics

Table 1 shows the total number of live births captured per each GDHS and the number of neonatal, infant, and under-five deaths. Over this period, five demographic and health surveys were conducted in Ghana for which a total of 16,474 live births (average 3,295 live births per GDHS) were captured. Total neonatal, infant, and under-five deaths captured over this period was 673 (average 135 deaths per GDHS), 1,013 (average 203 deaths per GDHS), and 1,378 (average 276 deaths per GDHS), respectively. The average rates of decline per year for neonatal, infant, and under-five mortality were 0.6, 1.0, and 2.1%, respectively.

**Table 1:** Neonatal, infant, and under-five deaths, 1988–2008 Ghana Demographic and Health Survey

	Total live births	Neonatal deaths	Infant deaths	Under-five deaths
	
GDHS	Number (n)	Number (rate)	Number (rate)	Number (rate)
GDHS 1988	4,136	198 (47.9)	299 (72.3)	446 (107.8)
GDHS 1993	2,204	94 (42.6)	130 (59.0)	148 (67.2)
GDHS 1998	3,298	109 (33.1)	192 (58.2)	272 (82.5)
GDHS 2003	3,844	166 (43.2)	235 (61.1)	314 (81.7)
GDHS 2008	2,992	106 (35.4)	157 (52.5)	198 (66.2)

Average rate of decline per year: neonatal mortality 0.6%, infant mortality 1.0%, and under-five mortality 2.1%. GDHS: Ghana Demographic and Health Survey.

### National trends in neonatal, infant, and under-five mortality

**Fig. 1:**
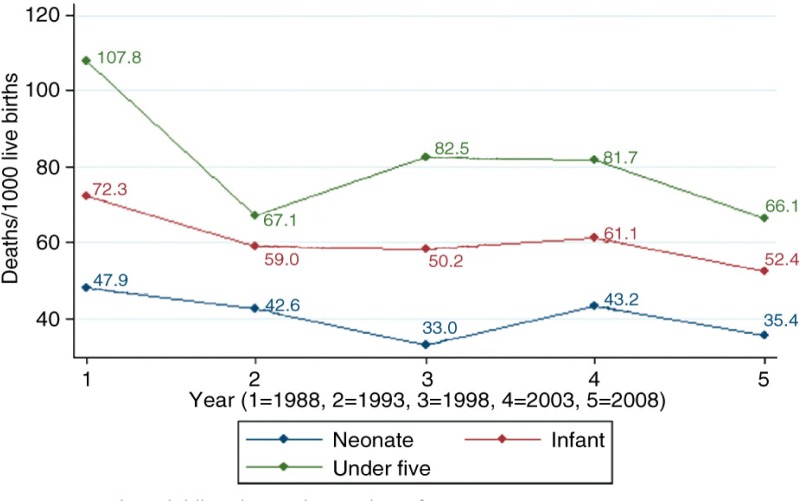
Trends in childhood mortality in Ghana from 1988 to 2008.

Figure 1 shows the trends in neonatal, infant, and under-five mortality from the 1988 to the 2008 GDHS while Table 2 reports the results of Poisson regression analysis that quantified the changes in the trends observed in Fig. 1. From 1988 to 1998, neonatal mortality declined from 47.9 per 1,000 to 33.1 per 1,000 and by 2008 neonatal mortality increased to 35.4 per 1,000. Considering the results in Table 2, neonatal mortality has not witnessed any significant decline over this period.

**Table 2:** Poisson regression analyses of the trend in neonatal, infant, and under-five mortality, 1988–2008 Ghana Demographic and Health Survey

	Neonatal death	Infant death	Under-five death
	
Year	IRR (95% CI)	IRR (95% CI)	IRR (95% CI)
1988 GDHS	1 (reference)	1 (reference)	1 (reference)
1993 GDHS	0.90 (0.60–1.35)	0.82 (0.58–1.15)	0.62 (0.46–0.84)**
1998 GDHS	0.69 (0.44–1.07)	0.81 (0.57–1.14)	0.76 (0.57–1.01)
2003 GDHS	0.90 (0.59–1.35)	0.85 (0.60–1.19)	0.76 (0.57–1.01)
2008 GDHS	0.73 (0.47–1.12)	0.72 (0.50–1.03)	0.61 (0.45–0.83)**

CI: confidence interval; GDHS: Ghana Demographic and Health Survey; IRR: incidence risk ratio;

** *p*<0.01.

Infant mortality declined from 72.3 per 1,000 to 58.2 per 1,000 from 1988 to 1998 and by 2008 infant mortality dropped to 52.5 per 1,000. However, the results in Table 2 shows that the decline observed in infant mortality from 1988 to 2008 was not statistically significant. From 1988 to 1998, under-five mortality declined from 107.8 per 1,000 to 82.5 per 1,000 and by 2008 under-five mortality had further declined to 66.2 per 1,000. Over the same period, the results in Table 2 shows that under-five mortality was significantly lower in 1993 and 2008 when compared with 1988. In 1993 and 2008, the risk of under-five death was reduced by 38% (IRR=0.62; 95% CI: 0.46–0.84) and 39% (IRR=0.61; 95% CI: 0.45–0.83), respectively, when compared with that of 1988.

**Fig. 2:**
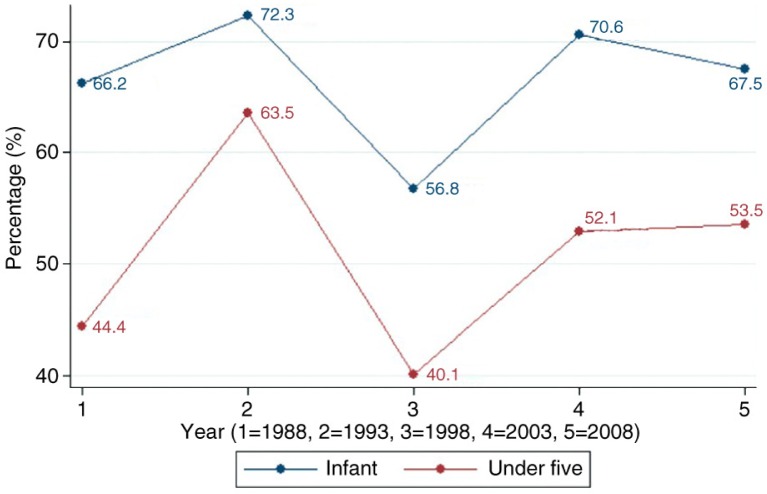
Trends in proportion of infant and under-five mortality attributed to neonatal death.

Figure 2 depicts the trends in the proportion of infant and under-five mortality attributable to neonatal deaths. From 1988 to 1998, the percentage of infant mortality attributed to neonatal mortality declined from 66 to 57%; however, by 2008, it increased to 67%. Likewise, from 1988 to 1998, the proportion of under-five mortality attributable to neonatal deaths reduced from 44 to 40%; however, by 2008 it increased to 53%.

### Regional trends in neonatal mortality

**Fig. 3:**
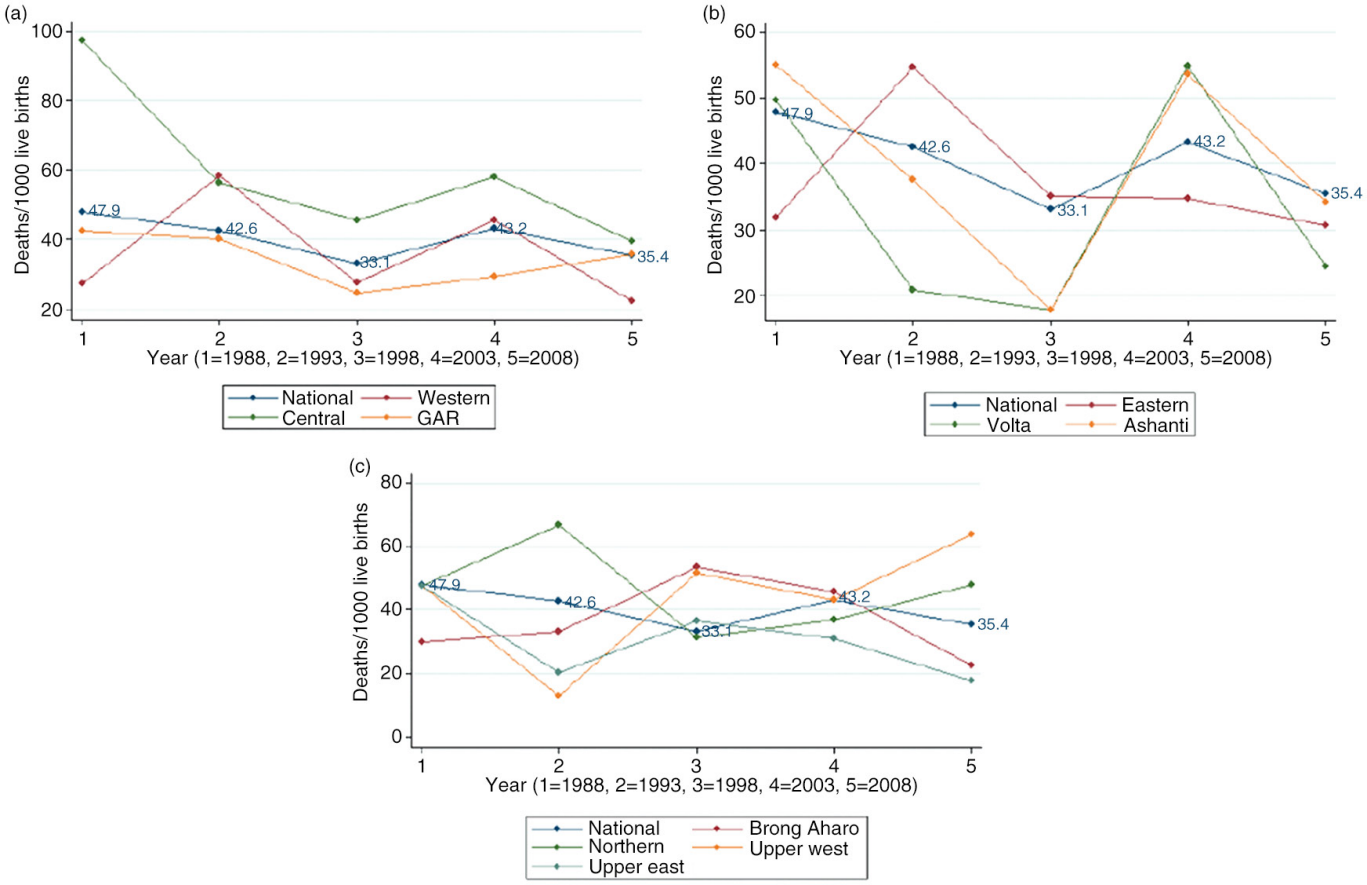
National and regional trends in neonatal mortality in Ghana.

The regional trends of neonatal mortality are shown in Fig. 3. In 1988, neonatal mortality rates in the Central, Volta, and Ashanti regions were above the national rate (48 neonatal deaths per 1,000 live births); in 1998, Central, Eastern, Brong Ahafo, Upper East, and Upper West regions had a higher neonatal rate than the national average (33 neonatal deaths per 1,000 live births). By 2008 the Central, Upper West, and Northern regions exceeded the national neonatal mortality rate (35 neonatal deaths per 1,000 live births). The neonatal mortality in the Central region was persistently higher than the national average, whereas neonatal mortality in the Greater Accra region (GAR) stayed below the national average from 1988 to 2008.

### Impact of implemented health policies and intervention programs on MDG 4 in Ghana

Following the adoption of the MGDs in Ghana, the Ghanaian government in collaboration with international donors implemented several intervention programs and health policies aimed at accelerating attainment of MDG 4 and MDG 5 in Ghana. We examined the impact and limitations of national health policies that were implemented post-adoption of MDG 4 until 2008. Findings of studies that have assessed the policies quantitatively and/ or qualitatively have been summarized in Table 3. At the national level, the LSS (7), SMP (6), and IMCI (8) programs were initiated between 1988 and 1998 and subsequently scaled up thereafter.

**Table 3:** Overview of National Health Policies implemented to address childhood mortality in Ghana from 1988 to 2008

National Health policy	Activities	Time of assessment	Findings of studies assessing the effectiveness of national health policy programs
Safe Motherhood (6) Program (SMP)	Ghana SMP entails primary health care, antenatal care, essential obstetric care, clean/safe delivery, family planning and equity for women. (Launched in 1993 and scaled up in 2000.)	After scale-up	Okiwelu et al. showed that some donors were implementing other interventions outside the objectives of the SMP, and the authors concluded that such action might dilute the expected effect of the policy (35).
			Anderson et al. identified migration of care providers (medical doctors) out of Ghana as one of the main factors that hampered the SMP in Ghana (36).
			Maine et al. in his review on the SMP showed that the policy was not well-defined and most policy makers believed that most of the components of SMP were already implemented prior to the SMP (37).
Community-Based Health Planning and Services (9) (CHPS)	Community health officer (CHO) provides the following services: treatment of minor illness, health education, family planning, skilled delivery, and antenatal and postnatal care. Community volunteers are trained to carry out community mobilization. (First piloted in 1999, adopted nationwide in 2005.)	Prior to scale-up (experimental phase)	Prior to policy implementation at the national level, Phillips et al. showed that the CHPS program decreased childhood mortality and fertility rate (24).
		Prior to scale-up	Prior to policy implementation at the national level, Debpuur et al. showed that the CHPS program increased women's knowledge of contraception, willingness for birth spacing, and usage of contraception (38).
			Before the policy was adopted nationally, Pence et al. showed that the CHPS program decreased childhood mortality (39).
			Before the CHPS program was adopted, Binka et al. found that the program decreased childhood mortality and improved parental health-seeking behavior (40).
			Phillips et al. observed that CHPS improved contraceptive usage before the policy was adopted nationwide (41).
			Prior to the adoption of the policy, Awoonor-Williams et al. showed that CHPS increased usage of contraception, skilled antenatal delivery, and postnatal attendants (42).
		During scale up,	During the scale-up phase, Awoonor-Williams et al. observed the following challenges: inadequate funding, less preparedness of community health officer, inadequate community engagement, shortage of manpower and equipment and inadequate monitoring (31).
		After the adoption of the policy	Assessment of the CHPS initiative by Adongo et al. after its adoption showed that the program improved the acceptance of family planning (43).
			Following adoption of the CHPS, Adongo et al. observed that the implementation of the program in urban areas was difficult due to contextual differences between rural (where the CHPS was tested) and urban areas, suggesting further modification of the implementation strategies (44).
		Post-adoption of CHPS initiative	During post-adoption of CHPS, Nyonator et al. identified the following: inadequate community engagement, lack of funds made health managers to perceive CHPS as an administrative burden (9).
User Fees Exemption for Delivery Care (UFEDC) (10)	Exemption for pregnant women from paying delivery fees in order to increase skilled delivery. Public, private, and mission health care providers were receiving reimbursement for service rendered (Initiated in 2003, scaled up in 2005)	Prior to scale up	Before the policy was adopted, Asante et al. reported that the policy decreased catastrophic out-of-pocket payment (45). Before the policy was scaled up, Bosu et al. showed that the policy had no statistically significant effect on maternal mortality (25). Before the scaling up of the policy, Penfold et al. observed that the policy increased skilled delivery and reduced inequality in the utilization of maternal healthcare service (46). McKinnon et al. observed that facility-based delivery increased while neonatal mortality decreased (47).
		After scale up	Witter et al. reported that the stakeholders believed that the policy was a cost-effective initiative that can reduce inequality in the utilization of maternal healthcare service. Insufficient funding, inadequate management, irregular reimbursement, increased workload without any increase in staff strength subsequently hampered the quality of maternal healthcare (29).
			Witter et al. reported that the stakeholders believed that the policy was a good initiative to improve skilled delivery. The study showed improvement in early antenatal registrants but regions were not well consulted in terms of reimbursement. Consequently, reimbursement was erratic and insufficient (30).
			The study conducted by Witter et al. showed that the policy was well accepted as an effective strategy to improve safe delivery; contents of the policy were clear but insufficient; erratic funding delayed inadequate reimbursement; increased workload without incentive or any corresponding increase in the number of care providers militate against the sustainability of the policy (34).
			Meessen et al. observed 1) Agenda setting: It was not clear whether the policy was adopted as a result of pressure from donors or taking the advantage of the offer of being a “low resource setting”. 2) Policy formulation: Assessment of this policy based on good practices in policy formulation showed that the objectives of the policy were clear and the stakeholders welcomed the policy but its formulation was not free from donor's influence. Important policy formulation good practices such as situation analysis, assessment of different policy options, and stakeholders’ involvement were not observed. 3) Implementation stage: suffered from erratic and insufficient funding (27).
Focused Antenatal Care (FANC) (11)	Individualized care for pregnant women to improve efficiency and safe delivery.It involves early detection of complication, pre-existing morbidity, birth preparedness, health education, and health promotion. For a healthy woman, four antenatal visits at <16, 26, 32, and 36 weeks were recommended. (Implemented in 2002)	During policy implementation	Increased antenatal registrants, increased early antenatal registrants, improved patient–doctor interaction, reduced waiting time, improved quality of antenatal care, increased health facility delivery, reduced stillbirth, and increased postnatal care utilization were observed by Deganus et al. following the implementation of FANC (26). Nyarko et al. reported that both patients and healthcare providers accepted the policy. It improved the quality of antenatal care. However, there was no difference between the intervention facilities and the control facilities in terms of birth preparedness, complication readiness, and postnatal care. In addition, some intervention facilities were unable to implement some of the components of FANC due to lack of equipment (33).
National Health Insurance Scheme (12) (NHIS)	National health insurance for pregnant women: six antenatal visits, delivery (incl. obstetrics complications), two postnatal visits within 6 weeks post-delivery, neonatal care up to age 3 months. (Implemented in 2008)	Following the implementation of NHIS	Witter et al. showed that the policy makers did not learn from errors of free delivery policy; NHIS policy formulation was top-down, politically induced by donors, no well-prepared policy guidelines, no proper consultation, poor communication of the policy, no proper costing, no additional funds were made available, no long-time financial plan, erratic and insufficient reimbursement. Sub-optimal implementation, lack of adequate monitoring and evaluation, increased workload with a negative impact on healthcare quality. Despite these limitations implementation of the NHIS increased access to healthcare (32).
Integrated Management of Childhood Illness (8) (IMCI)	Aims to improve case management at primary level of care, management of childhood illnesses, and family and community childcare practices. It involves antenatal, delivery, and postnatal services; treatment and prevention of infectious diseases (pneumonia, diarrhea, malaria, measles, HIV/AIDS); improves nutrition (improves breastfeeding, reduces malnutrition), vaccination, and psychosocial development. (Started in 1998, by 2000 all districts started IMCI.)	Following the implementation of IMCI	Baiden et al. observed that many of the care providers were yet to receive training on IMCI. The study showed a significant level of non-compliance with the IMCI guidelines; all the 11 items in the IMCI checklist were observed in just 1% of the children. 95% of them received antimalarial treatment but only 11% underwent laboratory investigation (28).

Maine et al. provided assessment was a general assessment of the SMP.

Additional interventions such as the CHPS (9), User Fees Exemption for Delivery Care (UFEDC) (10), FANC (11), and the NHIS (12) were implemented from 1999 onward to complement the impact of the existing programs so as to accelerate attainment MDG 4 and MDG 5. Maternal and child policies reviewed (Table 3) showed that most of the policies were directed at maternal, infant, and under-five mortality rather than neonatal mortality. Results presented indicate that these policies seem to have a greater effect on maternal healthcare utilization and maternal and childhood mortality and morbidity during the initiation phase than the scale-up phase (24–26, 48). Factors consistently identified to have a negative impact on the effectiveness of the various interventions were: deviation from good standard practice in policy formulation and implementation, erratic funding, insufficient community engagement, lack of proper monitoring, and inadequate manpower and equipment.

## Discussion

This study compared the trends in neonatal, infant, and under-five mortality from 1988 to 2008 in Ghana. It also identified the impact and challenges of various health policies and programs implemented during this time period to attain MDG 4. Despite the global attention on childhood mortality, we noticed that from 1988 to 2008 in Ghana, the decline rates in neonatal, infant, and under-five mortality were far below the expectation of a 4% annual decline to attain MDG 4 globally (49) and less than the 7% annual reduction stipulated to achieve MDG 4 in sub-Saharan Africa (50).

Similar to what Baiden et al. (51) and Welaga et al. (52) observed in the Kassena-Nankana district of Northern Ghana, the observed trends in childhood mortality cannot be directly attributed to the various overlapping policies and programs implemented. However, some important observations were noticed. Health policies and intervention programs implemented from 1988 to 1998 (SMP (6), LSS for midwives (7), and IMCI (8)) were observed to have a larger effect on childhood mortality than those implemented from 1998 to 2008 (UFEDC (10), Focus Antenatal Care (FANC) (11), NHIS (12), and CHPS (9)).

Generally, the decline rates in neonatal, infant, and under-five mortality were far below expectations, and the implemented health policies and intervention programs appeared to have had more impact on under-five mortality than on neonatal and infant mortality. Due to the paltry decline in neonatal mortality, the proportion of infant and under-five mortality attributed to neonatal mortality has increased; this mimics global and SSA observations (49, 53). In addition, we identified in our review factors that were responsible for the slow decline observed in neonatal, infant, and under-five mortality. Studies that have evaluated health policies and intervention programs implemented in Ghana repeatedly showed that factors such as deviation from good standard practice in policy formulation and implementation (27, 28), erratic funding (29, 30), insufficient community engagement (9), inadequate monitoring (31, 32), and inadequate manpower and equipment (29, 33) are major challenges of health policies and programs that might have hindered a more pronounced decline in childhood mortality. Our observation was corroborated by a previous multi-country study that identified factors such as inadequate policy formulation and implementation, poor financing, shortage of health human resources, lack of re-training of staff, inadequate medical products and technologies as the major constraints to scale up intervention programs to improve survival in early life (54).

At the regional level, we observed some degree of variation in neonatal mortality trends. This observation may partly be explained by differences in implementation of national health policies and programs in conjunction with the disparities in additional programs implemented in the regions; examples of such regional differences are the Kybele program in the Greater Accra region (13, 14), kangaroo mother care (55), UNICEF-sponsored ACSD (15) in Northern Ghana, High Impact Rapid Delivery (HIRD) (56), and Project Five Alive (57, 58). The variation may also be driven by differences in baseline rate of neonatal mortality across the regions.

### Recommendation

Considering the slow rate of decline in childhood mortality, particularly in neonatal mortality, implementation of cost-effective, neonatal-specific interventions, such as newborn resuscitation, exclusive breastfeeding, use of partograph, kangaroo mother care, use of micronutrients, tetanus toxoid immunization, will be needed to successfully address attainment of SDG 3.2 (2, 5, 59). In addition, implemented interventions to tackle childhood mortality should be reformed based on the recurrent defects identified in policy formulation and implementation to accelerate attainment of SDG 3.2 (9, 31, 34).

### Study limitations and strengths

This is the first study in Ghana that utilized nationally representative data to examine trends in childhood mortality, allowing us to generalize our findings. GDHS data are generally regarded as high-quality data because of the sampling technique and the excellent household and respondent response rates (22). We went beyond the traditional graphical description of the mortality trends by applying Poisson regression to quantify the risk of dying over time. However, we are aware that there may have been the possibility of underreporting and misclassification in childhood mortality as a result of recall bias (60). In addition, non-sampling error such as misunderstanding of the question on the part of the participant or the interviewer could have occurred. As the current study was based on published articles, some valuable information on the impact and challenges of the intervention programs implemented and reported in the grey literature may not have been fully captured in this study. Also, most articles that assessed the implemented intervention programs were not properly designed to evaluate the effectiveness of these intervention programs.

## Conclusion

This study compared the trends in neonatal, infant, and under-five mortality over two decades in Ghana. The observed decline rates were generally slow, particularly for neonatal mortality. This could be attributed to the short-comings identified for health policies and intervention programs formulation and implementation, particularly with regard to neonatal mortality. Implementation of a sustainable evidence-based neonatal-specific intervention and improving other existing interventions will be a prerequisite to actualize SDG 3.2 in Ghana and beyond.

## Summary

What's known: Interventions were implemented in Ghana to achieve MDG 4 but the impact and challenges have not been assessed to inform SDG 3.2.

What's new: Since 1988, the decline in childhood mortality in Ghana was below the expected rate and the proportion of infant and under-five mortality attributed to neonatal death has increased because implementation of most intervention programs was suboptimum and newborns less considered. Implications: Implement neonatal-specific interventions and improve existing programs.

## Paper context

Since the adoption of Millennium Development Goal 4 in Ghana to reduce under-five mortality by two-thirds between 1990 and 2015, efforts were made towards attainment. However, impacts and challenges of implemented intervention programs have not been examined to inform implementation of SDG 3.2. This study examined what has been achieved in neonatal, infant and under-five mortality over two decades and highlighted the impacts and challenges of intervention programs implemented. This study provided important information that policy makers can utilize towards actualizing SDG 3.2.
